# PRKCQ promotes oncogenic growth and anoikis resistance of a subset of triple-negative breast cancer cells

**DOI:** 10.1186/s13058-016-0749-6

**Published:** 2016-09-23

**Authors:** Jessica Byerly, Gwyneth Halstead-Nussloch, Koichi Ito, Igor Katsyv, Hanna Y. Irie

**Affiliations:** 1 Division of Hematology and Medical Oncology, Department of Medicine and Department of Oncological Sciences, Tisch Cancer Institute, Icahn School of Medicine at Mount Sinai, 1468 Madison Avenue, New York, NY USA; 2 Department of Oncological Sciences, Tisch Cancer Institute, Icahn School of Medicine at Mount Sinai, 1468 Madison Avenue, New York, NY USA; 3 Department of Genetics and Genomic Sciences, Icahn School of Medicine at Mount Sinai, New York, NY 10029 USA

**Keywords:** PRKCQ/PKCθ, Triple-negative breast cancer, Anoikis, EGF-independent growth

## Abstract

**Background:**

The protein kinase C (PKC) family comprises distinct classes of proteins, many of which are implicated in diverse cellular functions. Protein tyrosine kinase C theta isoform (PRKCQ)/PKCθ, a member of the novel PKC family, may have a distinct isoform-specific role in breast cancer. PKCθ is preferentially expressed in triple-negative breast cancer (TNBC) compared to other breast tumor subtypes. We hypothesized that PRKCQ/PKCθ critically regulates growth and survival of a subset of TNBC cells.

**Methods:**

To elucidate the role of PRKCQ/PKCθ in regulating growth and anoikis resistance, we used both gain and loss of function to modulate expression of PRKCQ. We enhanced expression of PKCθ (kinase-active or inactive) in non-transformed breast epithelial cells (MCF-10A) and assessed effects on epidermal growth factor (EGF)-independent growth, anoikis, and migration. We downregulated expression of PKCθ in TNBC cells, and determined effects on in vitro and in vivo growth and survival. TNBC cells were also treated with a small molecule inhibitor to assess requirement for PKCθ kinase activity in the growth of TNBC cells.

**Results:**

PRKCQ/PKCθ can promote oncogenic phenotypes when expressed in non-transformed MCF-10A mammary epithelial cells; PRKCQ/PKCθ enhances anchorage-independent survival, growth-factor-independent proliferation, and migration. PKCθ expression promotes retinoblastoma (Rb) phosphorylation and cell-cycle progression under growth factor-deprived conditions that typically induce cell-cycle arrest of MCF-10A breast epithelial cells. Proliferation and Rb phosphorylation are dependent on PKCθ-stimulated extracellular signal-related kinase (Erk)/mitogen-activated protein kinase (MAPK) activity. Enhanced Erk/MAPK activity is dependent on the kinase activity of PKCθ, as overexpression of kinase-inactive PKCθ does not stimulate Erk/MAPK or Rb phosphorylation or promote growth-factor-independent proliferation. Downregulation of PRKCQ/PKCθ in TNBC cells enhances anoikis, inhibits growth in 3-D Matrigel^TM^ cultures, and impairs triple-negative tumor xenograft growth. AEB071, an inhibitor of PKCθ kinase activity, also inhibits growth and invasive branching of TNBC cells in 3-D cultures, further supporting a role for PKCθ kinase activity in triple-negative cancer cell growth.

**Conclusions:**

Enhanced PRKCQ/PKCθ expression can promote growth-factor-independent growth, anoikis resistance, and migration. PRKCQ critically regulates growth and survival of a subset of TNBC. Inhibition of PKCθ kinase activity may be an attractive therapeutic approach for TNBC, a subtype in need of improved targeted therapies.

**Electronic supplementary material:**

The online version of this article (doi:10.1186/s13058-016-0749-6) contains supplementary material, which is available to authorized users.

## Background

Triple-negative breast cancer (TNBC) represents approximately 15 % of all breast cancer and is a subtype for which therapeutic options are more limited due to lack of effective targeted therapies. Triple-negative breast cancer is defined by the lack of expression of estrogen receptor (ER), progesterone receptor (PR), and human epidermal growth factor receptor 2 (Her2), but it is genetically and genomically diverse. Due to the lack of targeted therapies and intrinsic biological aggressiveness, novel therapeutic strategies, beyond standard-of-care chemotherapy, are desperately needed.

We first identified protein tyrosine kinase C theta isoform (PRKCQ)/PKCθ as a candidate regulator of anchorage-independent survival of breast cancer cells in a functional kinome screen [1]. PRKCQ/PKCθ is a member of the novel protein kinase C (PKC) family characterized by a unique protein domain structure consisting of diacylglycerol binding sites, but lacking calcium (Ca+) binding sites typical of classical PKCs. PRKCQ maps to Chromosome 10p15, a region frequently mutated in T cell leukemia, lymphoma and T cell immunodeficiency (as reviewed in [2]). PRKCQ/PKCθ is widely expressed throughout the hematopoietic system, primarily in T cells, mast cells, natural killer (NK) cells and platelets, and in skeletal muscle, liver, thymus, and the nervous system [3–6].

Much of the known isoform-specific functions of PRKCQ/PKCθ are in the context of immune function; mice deficient in PRKCQ expression exhibit defects in T cell activation due to impaired Ca+  signaling and nuclear factor of activated T cells (NFAT) activation [7, 8]. PRKCQ/PKCθ also regulates the survival of T cells by regulating the expression of pro-apoptotic and anti-apoptotic B cell lymphoma 2 (Bcl2) family members [2, 8, 9]. More recent evidence supports a relative specific role for PRKCQ in immune response; it is mostly dispensable for immunity against viral and bacterial pathogens [2, 10–12]. In contrast, PRKCQ appears to be required for immune responses associated with autoimmune disease and allograft rejection, perhaps due to a specific requirement for PRKCQ in the maturation of T helper (Th)17 cells, a subset of CD4+ T cells [13–16].

In contrast, the functions of PRKCQ in non-hematopoietic tissue and in cancer have not yet been fully elucidated. PRKCQ is reported to be expressed in solid tumors including gastrointestinal stromal tumors (GIST) and more recently, in breast cancer, specifically ER-negative tumors [17–20]. In fact, PRKCQ expression directly suppresses the expression of ERα in breast cancer cells and is required for c-rel-induced mammary tumorigenesis [20]. PRKCQ also stimulates breast cancer cell migration by stabilizing the expression of Fra-1 in TBNC cells [21]. Furthermore, PRKCQ may contribute to the formation or maintenance of a breast cancer stem cell population by promoting the expression of genes associated with epithelial-mesenchymal transition (EMT) through direct chromatin interactions [22, 23].

The role of PRKCQ/PKCθ in the proliferation and survival of breast cancer cells and the responsible mechanisms, including dependency on kinase activity, remain to be clarified. Here we show that PRKCQ is sufficient to drive growth-factor-independent proliferation, migration and anoikis resistance of breast epithelial cells (MCF-10A). PRKCQ promotes proliferation by activating extracellular signal-related kinase (Erk)/mitogen-activated protein kinase (MAPK) activity in a kinase-activity-dependent manner. PRKCQ is not only sufficient to promote these phenotypes in MCF-10A cells, but, we showed for the first time that it is required for in vitro and in vivo growth of a subset of TNBC cells. These studies support PRKCQ/PKCθ as an attractive candidate therapeutic target in TNBC.

## Methods

### Reagents, cells, and cell culture

MDA-MB-231-luc-D3H2LN cells were obtained from Perkin Elmer. Cells were cultured in MEM with Earle’s Salts supplemented with non-essential amino acids, GlutaMAX™, sodium pyruvate, penicillin/streptomycin (P/S; Life Technologies) and 10 % heat-inactivated fetal bovine serum (FBS) (Life Technologies) at 37 °C in 5 % CO2. All other cell lines were purchased from ATCC (Manassas, Virginia). MDA-MB-436 cells were cultured in Leibovitz’s L-15 media supplemented with 10 ug/mL insulin, 16 ug/mL glutathione, P/S, and 10 % FBS at 37 °C under atmospheric conditions. MCF10A cells were cultured in 1:1 DMEM/F12 supplemented with 5 % horse serum, 20 ng/mL EGF (Peprotech), 500 ug/mL hydrocortisone (Sigma-Aldrich), 100 ng/mL cholera toxin (Sigma-Aldrich), 10 ug/mL insulin (Sigma-Aldrich), and penicillin/streptomycin (Life Technologies). HCC1806 and HCC38 cells were cultured in RPMI-1640 media supplemented with 10 % FBS and P/S. AEB071 was purchased from Selleckchem.

Antibodies directed against the following proteins were obtained from the indicated suppliers: AbCam: rabbit monoclonal protein kinase C (PKC-θ) (EPR1487(2)); Cell Signaling Technologies: phospho-retinoblastoma (Rb) (S807/811) and total Rb, phospho-p44/p42 MAPK (ERK1/2) (T202/Y204) and total p44/42 MAPK (ERK1/2), phospho-Akt (S473) and total (pan-) Akt, PKC isoform antibody sampler kit (for PKCδ, PKCα, PKCμ, PKCζ) phospho-PKCθ (T538), phospho-PKCα/βII (T638/641), glyceraldehyde-3-phosphate dehydrogenase (GAPDH), and beta-tubulin. Additional GAPDH antibody from Santa Cruz Biotechnology was used.

### Constructs, viral production, and stable cell line generation

PRKCQ complementary DNA (cDNA) encoding retroviral vector was obtained from Joshua LaBaer (Harvard Institute of Proteomics). Mutagenesis to create PRKCQ mutants was performed using QuikChange Lightning Site-Directed Mutagenesis Kit (Stratagene) and the following primers: 5′ GCATCAGCGCCGGGGTGAAATCAAGCAGGCAAAGGTC-3′ for A148E (constitutively active) and 5′ CCAATCAATTTTTCGCAATAAGGGCCTTAAAGAAAGATGTGG-3′ for K409R (catalytically inactive) [24]. Retrovirus was generated using 293-GPG cells (gift of R. Mulligan) and according to protocols described previously [25].

Constructs encoding short hairpin RNA (shRNA) sequences targeting PRKCQ (TRCN0000001790, TRCN0000199654 and TRCN0000197216, referred to as 90, 54 and 16, respectively) were purchased from Open Biosystems/Thermo Scientific. Viral packaging 293 T cells were transfected according to standard protocols to produce lentiviral particles, as described previously [25]. Viral supernatant was collected 24, 48, 72, and 96 hours post-transfection, pooled, and concentrated using Amicon Ultra Centrifugal Filter Units (Millipore). MDA-231-luc or MDA-436 cells were spin-infected in the presence of 2 ug/mL polybrene (Sigma-Aldrich) and concentrated viral supernatant at 2250 rpm for 30 minutes. Cells were exposed to viral supernatant overnight before changing to complete media and were allowed to recover for 24 hours.

### Anoikis and transwell migration assays

Anoikis (anchorage-independent viability) assay was performed by culturing cells in suspension on polyhema-coated plates for the indicated amount of time, and cell death was assessed using the Cell Death ELISA^PLUS^ Kit (Roche) according to manufacturer’s instructions. Transwell migration assay was performed as described previously [25].

### Proliferation assay/growth curves

Cells were plated at a density of 1 × 10^4 cells per well in triplicate in a 24-well plate. Cells were harvested and counted using a hemacytometer at the indicated number of days after seeding.

### 3-D culture

Three-dimensional (3-D) Matrigel™ (BD Biosciences) cultures were performed as described previously using 3000–5000 cells/well [25].

### Cell cycle analysis/flow cytometry

Cells were plated at subconfluent densities, harvested using 0.05 % Trypsin/EDTA (Life Technologies), washed with PBS, resuspended as single cells in cold PBS and fixed with ice-cold ethanol at a final concentration of 70 %. After fixation cells were stored at 4 °C until staining. Cells were stained using propidium iodide (PI)/RNase Staining Buffer (BD Biosciences) at 37 °C for 30 minutes before sample processing. DNA content was assessed by PI staining using BD Canto and FACSDiva. Cell cycle profiles were analyzed using Flow Jo (version 9.0). Percentages were calculated using the Watson pragmatic algorithm. Significance was calculated using the standard Student *t* test.

### In vivo tumor xenograft models

Female nude mice (nu-/-) were obtained from Jackson Laboratories. At age 6–8 weeks, 5 × 10^5 MDA-231-luc cells per mouse were injected subcutaneously in a total volume of 100 uL of complete media 48 hours after infection with PRKCQ shRNA lentiviral particles. Tumor dimensions were measured with calipers and the volume was calculated as (L x W^2^)/2. Stastical significance was calculated using the Whitney-Mann-Wilcoxon rank sum test. All procedures and studies with mice were performed in accordance with protocols pre-approved by the Institutional Animal Care and Use Committee of Mount Sinai.

### PRKCQ transcript expression analysis in breast tumors

#### The Cancer Genome Atlas (TCGA) dataset

Level-3 expression IlluminaHiSeq-RNASeqV2 expression data were downloaded from the TCGA data portal [26] and processed for quality control as follows: log(x + 1) transformation was performed to rescale the expression data, followed by quantile-normalization, using normalize.quantiles() from R package “preprocessCore”. The quantile-normalized data were split for tumor and normal tissue samples. Correction for batch effects was performed using batch ID, tissue source site ID, center ID and plate ID, where batch ID was obtained from TCGA biospecimen files, and other IDs were obtained from TCGA barcode. Batch and age corrections were performed using the linear regression (lm()) function in the statistical computing software R, for each gene expression profile, thereby removing discrepancy between different batch IDs, and preserving the overall mean across all samples. Expression of PRKCQ was then extracted and patients were classified as receptor positive (ER, PR, or Her2 positive, *n* = 731) or TNBC (ER, PR, and Her2 negative, *n* = 86). The significance of difference in log expression was tested using the one-sided Student *t* test.

#### METABRIC dataset

METABRIC-normalized Illumina HT12v3 data were downloaded from the European Bioinformatics Institute, quantile-normalized, and corrected for age [27]. Samples were stratified as TNBC or receptor-positive as follows: samples with negative expression of ER, PR, and Her2, as reported by Curtis et al. [27] in the columns “ER.Expr,” “PR.Expr”, and “Her2.Expr,” respectively, and not classified as luminal A, luminal B, or Her2 by PAM50 subtyping, also reported by Curtis et al. [27], were labeled TNBC (n = 276); all other samples were labeled receptor-positive (n = 1698). PRKCQ expression was extracted and log expression was compared in the TNBC and receptor-positive samples using the one-sided Student *t* test.

### Consent statement

We confirm that this study does not involve human patients and no consent was necessary.

## Results

### PRKCQ is sufficient to promote anoikis resistance, migration and growth factor-independent proliferation

**Fig. 1:**
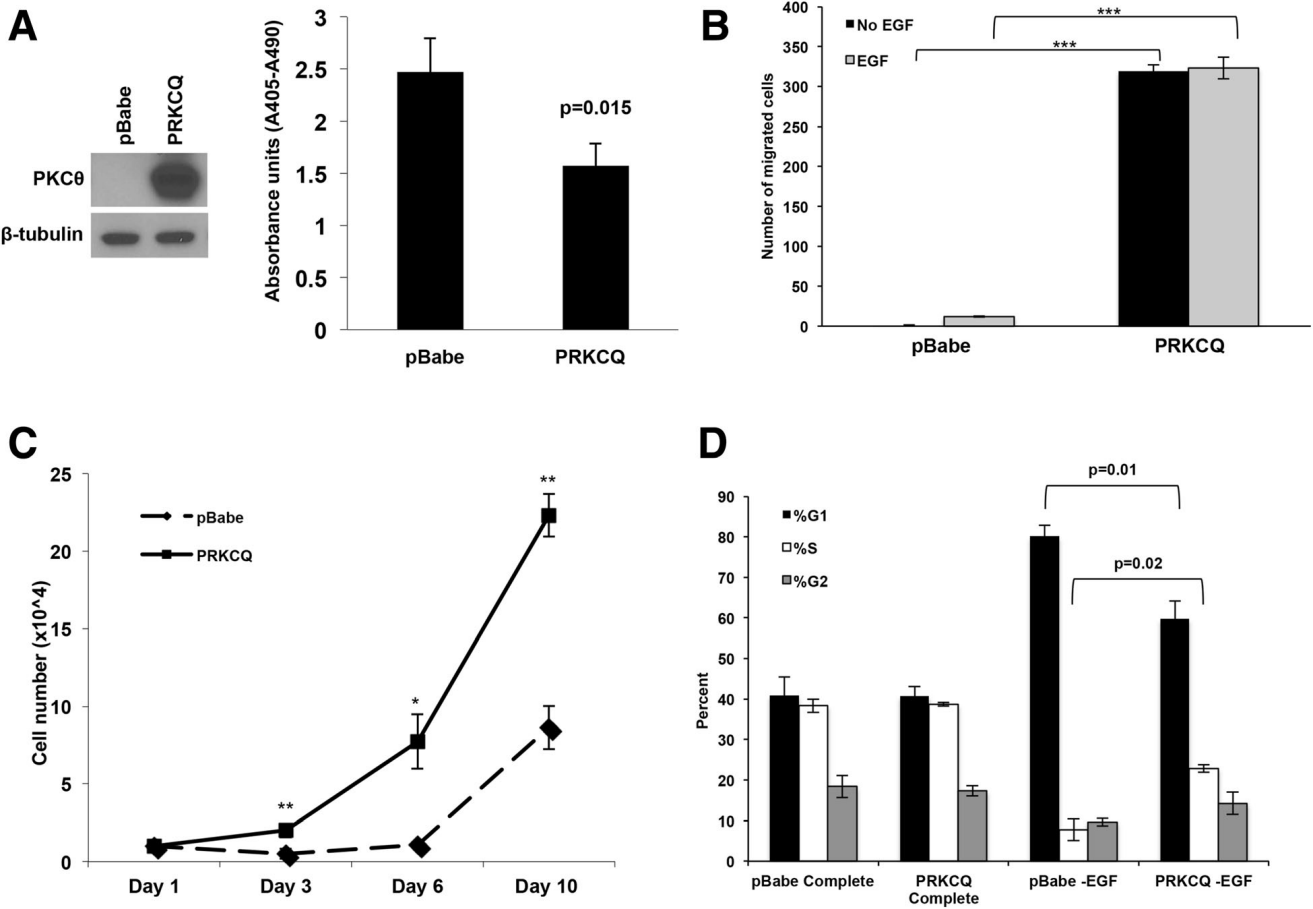
Protein tyrosine kinase C theta isoform (*PRKCQ*) overexpression promotes multiple oncogenic activities in breast epithelial cells. **a** Non-transformed, immortalized MCF-10A cells overexpressing vector control or PRKCQ were generated, and assessed for their ability to survive in suspension cultures using the Cell Death ELISA assay. **b** The ability of MCF-10A cells overexpressing vector control or PRKCQ in Transwell Boyden chamber assays in the presence or absence of epidermal growth factor (*EGF*) was evaluated. The number of cells that migrated 18 hours after plating in the insert was counted. Five fields of migrated cells (×10 objective) were counted and averaged. **c** MCF-10A cells expressing vector control or PRKCQ (1 × 10^4 cells) were plated on day 1 in growth media lacking EGF. The number of cells were counted on the indicated days and plotted. **d** Cell-cycle profile analysis of empty vector or PRKCQ-expressing cells was performed by fluorescence-activated cell sorting after propidium iodide staining of cells cultured in growth media +/- EGF: *p < 0.05, **p < 0.01, ***p < 0.001. *PKC* protein kinase C

During tumorigenesis, cells often acquire the ability to survive and grow in conditions (e.g., matrix or growth factor deprivation) that do not support proliferation of normal cells. For example, non-transformed, immortalized MCF10A breast epithelial cells are highly dependent on the presence of growth factors (e.g., insulin and EGF) for cell division and growth; absence of these growth factors in the culture medium induces cell-cycle arrest ([28] and Fig. 1d). To determine whether PRKCQ/PKCθ is sufficient to promote oncogenic activity, such as growth factor-independent growth, we expressed PRKCQ in MCF-10A cells, which express relatively low levels of basal PRKCQ. When overexpressed in MCF-10A cells, PRKCQ suppresses anoikis in suspension cultures (Fig. 1a) and enhances EGF-independent migration in Transwell assays (Fig. 1b). Furthermore, PRKCQ promotes EGF-independent growth in monolayer cultures; this is due to the ability of PRKCQ to partially prevent G1 arrest and promote cell-cycle progression in the absence of EGF stimulation (Fig. 1c and d). Therefore, PRKCQ is sufficient to enhance EGF-independent growth, anoikis resistance and migration of non-transformed MCF-10A breast epithelial cells.

### PRKCQ enhances EGF-independent growth by activating Erk/MAPK and Rb phosphorylation in a kinase activity-dependent manner

**Fig. 2:**
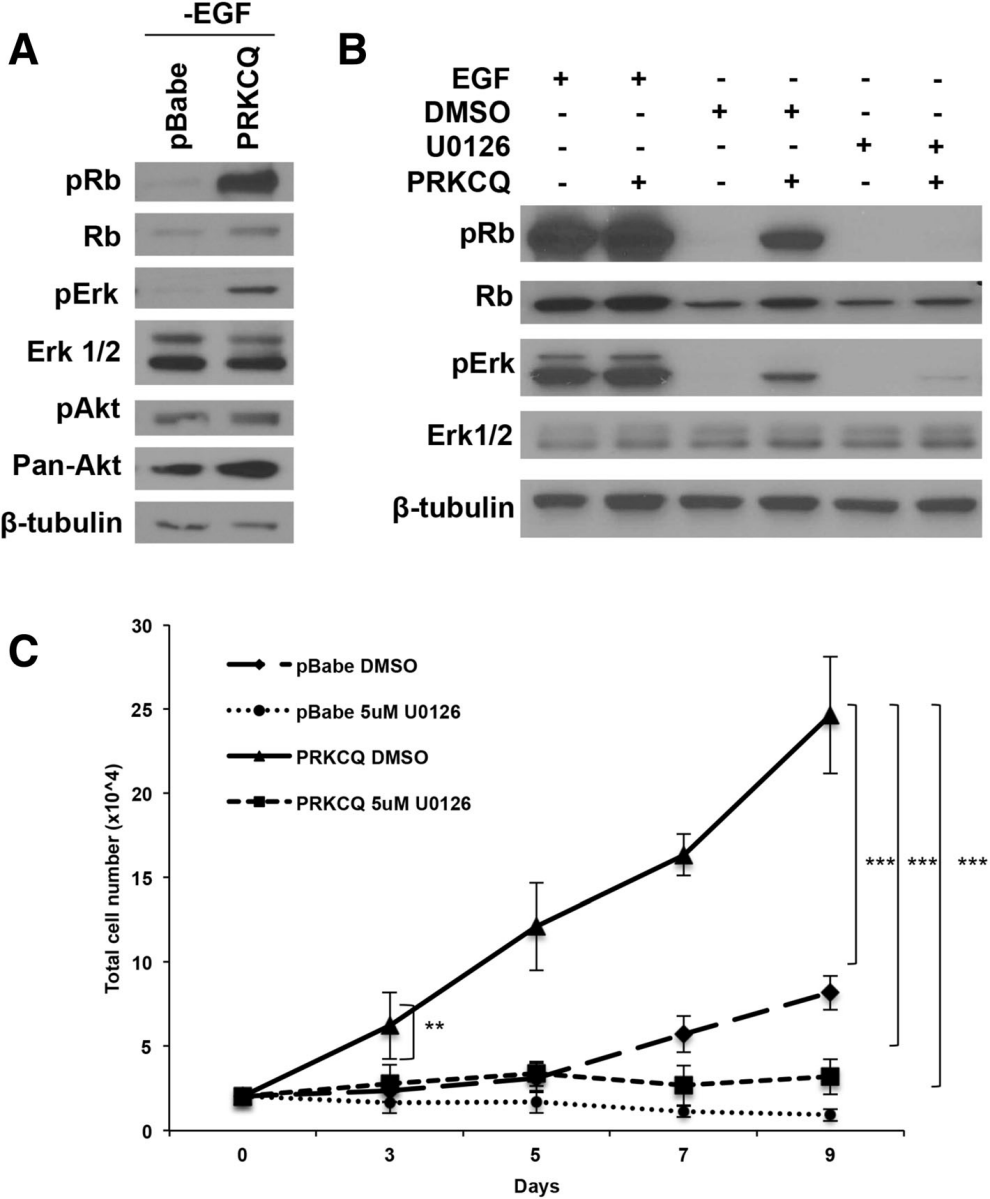
Extracellular signal-related (*Erk*)/mitogen-activated protein kinase (MAPK) activation is required for protein tyrosine kinase C theta isoform (*PRKCQ*)-mediated epidermal growth factor (*EGF*)-independent growth. **a** MCF-10A cells overexpressing pBabe vector control or wild-type PRKCQ were cultured in growth media lacking EGF. Lysates were probed with the indicated antibodies. **b** PRKCQ or vector control expressing MCF-10A cells were cultured in growth media +/- EGF. Cells grown in the absence of EGF were treated for 24 hours with dimethyl sulfoxide (*DMSO*) or mitogen-activated protein kinase kinase (MEK) inhibitor U0126 (5 μM). Cells were lysed and probed with the indicated antibodies. **c** MCF-10A cells expressing vector control or PRKCQ were cultured in growth media lacking EGF and treated with DMSO or U0126 (5 μM) for the indicated number of days. DMSO or U0126 was replaced every 2–3 days. Cell numbers were counted on the indicated days and plotted. *Rb* retinoblastoma

We sought to determine the mechanisms by which PRKCQ expression promotes cell-cycle progression under conditions that typically induce G1 cell cycle arrest of MCF-10A cells (e.g., EGF deprivation). Cells overexpressing PRKCQ/PKCθ exhibit enhanced levels of Rb phosphorylation when compared to vector control cells cultured in media lacking EGF. We examined the status of signaling pathways linked to cell-cycle progression and proliferation. Levels of activated Erk/MAPK, but not Akt, were increased in PRKCQ-overexpressing cells. (Fig. 2a). Erk/MAPK activity is required for PRKCQ-stimulated Rb phosphorylation and cell-cycle progression under EGF-deprived conditions; treatment of PRKCQ-overexpressing cells with U0126, an inhibitor of MAPK kinase (MEK), abrogated the increased Rb phosphorylation and the EGF-independent growth promoted by PRKCQ expression (Fig. 2b and c).

**Fig. 3:**
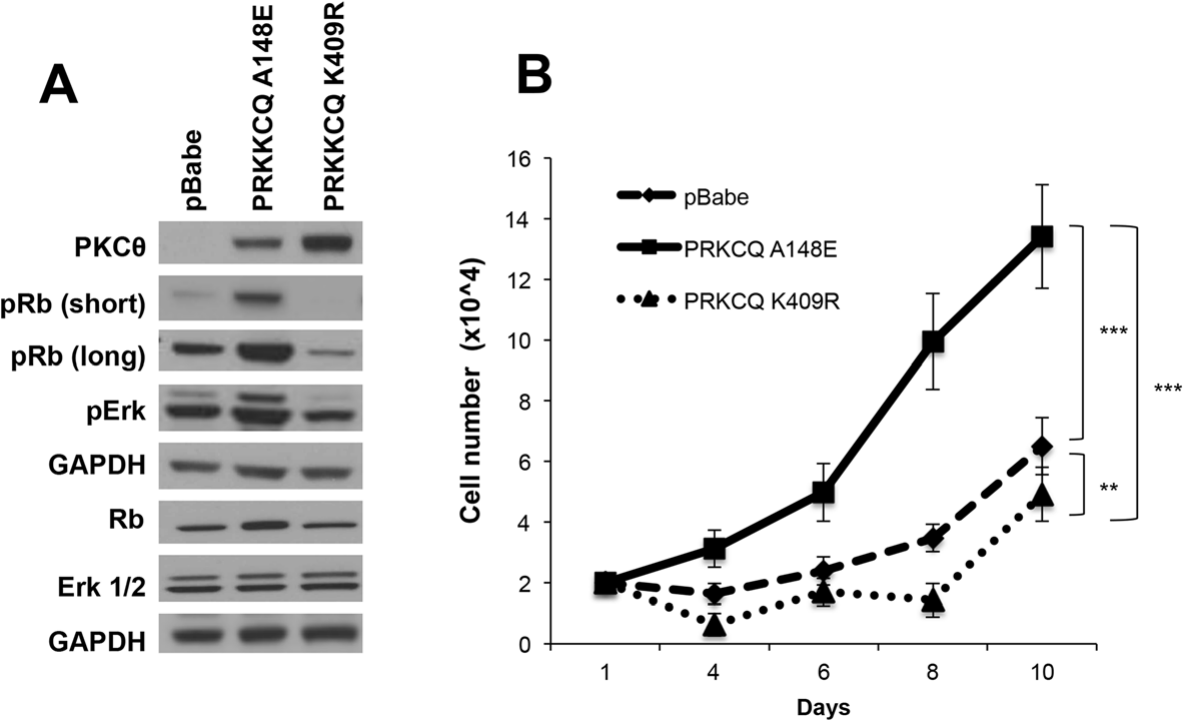
Protein tyrosine kinase C theta isoform (*PRKCQ*) kinase activity is required for epidermal growth factor (*EGF*)-independent growth, Extracellular signal-related kinase (*Erk* activity) and retinoblastoma (*Rb*) phosphorylation. **a** MCF-10A cells expressing vector control, constitutively active (A148E) or kinase-inactive (K409R) PKCθ, were cultured in growth media lacking EGF. Cell lysates were probed with the indicated antibodies. **b** MCF-10A cells expressing vector control, active or kinase-dead PKCθ, were cultured in media lacking EGF for the indicated number of days and counted

To determine if PRKCQ-stimulated Erk/MAPK activity, Rb phosphorylation, and cell-cycle progression are dependent on PRKCQ kinase activity, we generated both constitutively active (A148E) and kinase inactive (K409R) versions of PKCθ [24]. Overexpression of kinase-active PKCθ enhanced Erk/MAPK activity, Rb phosphorylation and EGF-independent growth, whereas overexpression of kinase-inactive PKCθ did not (Fig. 3a, b). These results support a critical role for kinase activity in PRKCQ-stimulated, EGF-independent Erk/MAPK activity, Rb phosphorylation and cell-cycle progression in MCF-10A cells.

### PRKCQ is preferentially expressed in TNBC

**Fig. 4:**
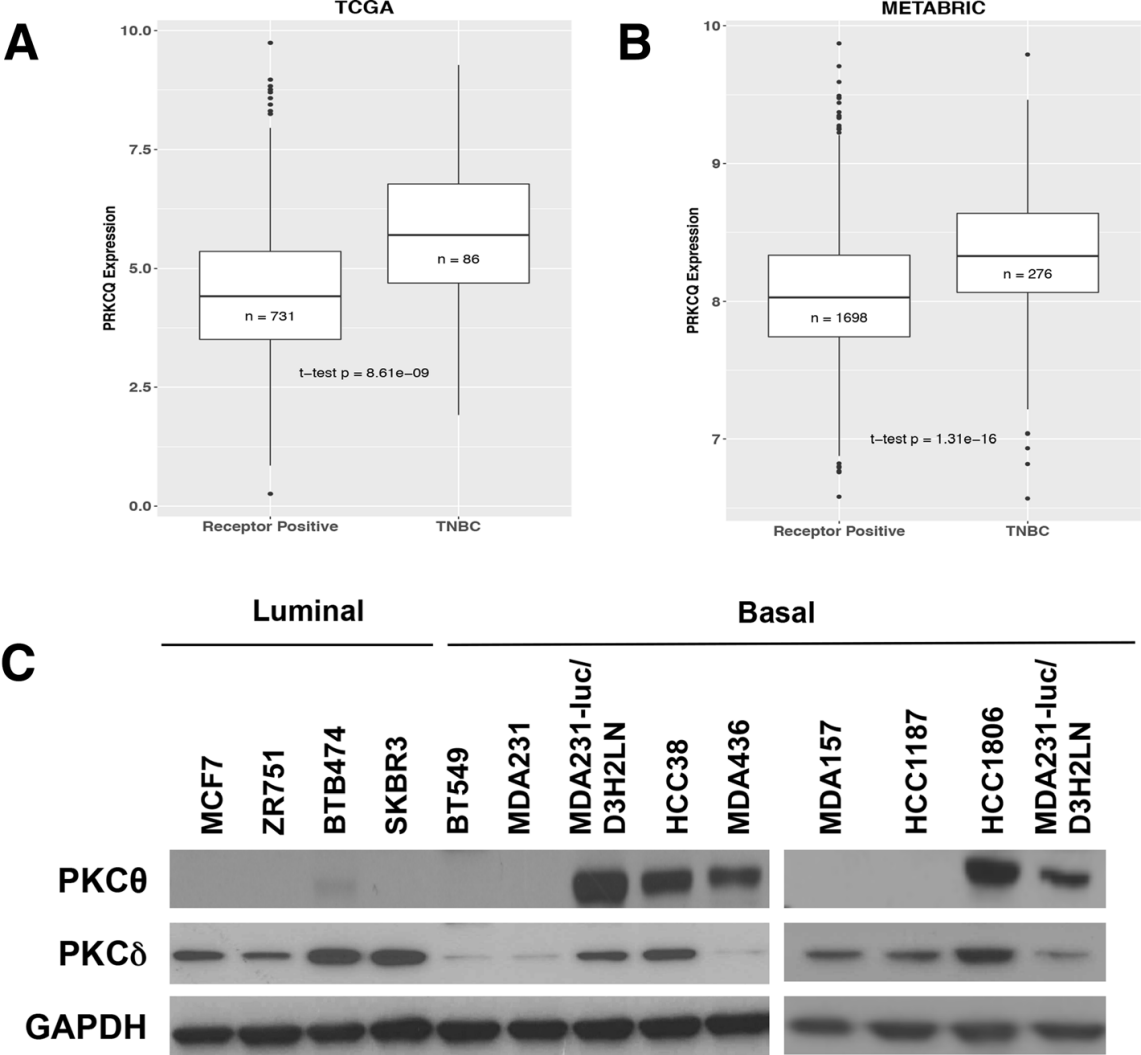
Protein tyrosine kinase C theta isoform (*PRKCQ*) is preferentially expressed in the triple-negative breast cancer (*TNBC*) subtype and breast cancer cell lines. PRKCQ transcript levels in triple-negative and hormone-receptor-positive or human epidermal growth factor (Her2)-positive patient tumors in The Cancer Genome Atlas dataset (*TCGA*) (**a**) and METABRIC dataset (**b**) were compared. **c** PKCθ protein expression in breast cancer cell lines was assessed by western analysis. Expression of PRKCδ, another novel PKC isoform, was also examined. *GAPDH* glyceraldehyde-3-phosphate dehydrogenase

We examined the level and pattern of expression of PRKCQ in patients’ breast tumors. Among tumors in TCGA dataset, PRKCQ transcript levels are higher in triple-negative tumors (e.g., negative for expression of ER, PR or Her2), when compared to tumors that express ER, PR and/or Her2 (*p* = 8.61 × 10^-9) (Fig. 4a). A similar pattern of higher PRKCQ transcript expression in patients’ triple-negative tumors compared to receptor-positive tumors was observed in the larger METABRIC dataset, which consists of nearly 2000 patient tumors (Fig. 4b). This expression pattern for PRKCQ is in agreement with a previous study that reported higher levels of PRKCQ transcript expression in ER-negative breast cancers when compared to ER-positive tumors, using other publicly available datasets [20]. This pattern of expression is also reflected in a panel of breast cancer cell lines that represent distinct breast cancer subtypes. Expression of PKCθ protein was detected in a subset of triple-negative, basal breast cancer cell lines, whereas ER-positive/luminal and Her2-positive breast cancer cell lines express none to barely detectable levels of PKCθ protein (Fig. 4c). Other novel PKC isoforms such as PKCδ did not have a similar differential expression pattern (Fig. 4c).

### Inhibition of PRKCQ impairs in vitro and in vivo growth of TNBC cells

**Fig. 5:**
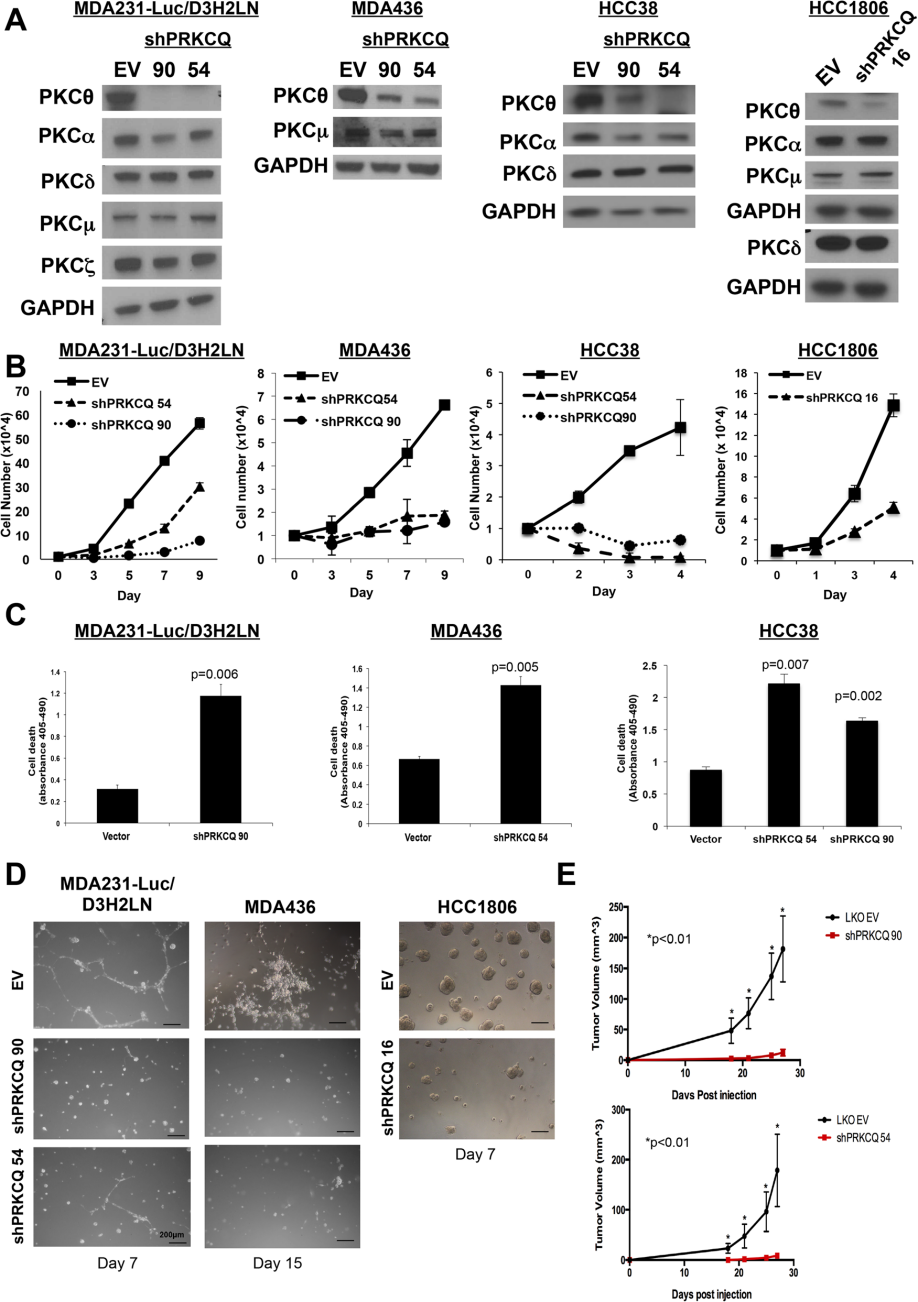
Protein tyrosine kinase C theta isoform (*PRKCQ*) downregulation inhibits growth of triple-negative breast cancer (TNBC) cells in culture and triple-negative breast tumor xenografts. **a** Lentiviral PRKCQ short hairpin (*sh*)RNA vector-infected TNBC cells were lysed and probed for expression of protein kinase C (*PKC*) family members. **b** TNBC cells expressing empty vector control (*EV*) or PRKCQ shRNA (90, 54 or 16) were grown in monolayer cultures for the indicated number of days and counted. **c** TNBC cells expressing vector control or PRKCQ shRNA (90, 54 or 16) were cultured in suspension for 24 hours. Cell death (anoikis) was assessed using the Cell Death ELISA. **d** TNBC cells expressing EV control or PRKCQ shRNA vectors were cultured in 3-D Matrigel^TM^ cultures in chamber slides for the indicated number of days. **e** MDA-231-Luc-D3H2LN cells (5 × 10^5^) expressing vector control or PRKCQ shRNA (90 or 54) were injected subcutaneously into the flank of 6-week-old, female nude mice. Tumor size was measured and recorded every 3–4 days. All figures are representative of three independent experiments except for the xenograft study, which was performed twice. *Scale bars* represent 200 μM. *GAPDH* glyceraldehyde-3-phosphate dehydrogenase

To begin to address the requirement for PRKCQ/PKCθ expression for growth and survival of TNBC cells, we downregulated its expression in cell lines with detectable levels of PKCθ protein (MDA-231-LucD3H2LN, MDA-436, HCC38, and HCC1806 cells) using several independent shRNA vectors (Fig. 5a). shRNA vectors that specifically downregulated expression of PKCθ, with minimal effect on expression of other PKC family members were chosen for functional studies. For the HCC1806 cell line, PRKCQ shRNA vector 16 was used, as vectors 90 and 54 resulted in significant downregulation of other PKC isoforms (Fig. 5a and unpublished data). In all cell lines evaluated, PKCθ protein downregulation severely compromised growth in 2-D monolayer cultures (Fig. 5b). The growth inhibition was consistent with arrest in the G2-M transition (Additional file 1: Figure S1). PKCθ downregulation in these TNBC cells also enhanced anoikis in suspension cultures (Fig. 5c). In 3-D Matrigel™ cultures, PKCθ downregulation inhibited growth, and the formation of invasive branches characteristic of TNBC cell lines (Fig. 5d). Importantly, infection of MDA-231 cells, which have undetectable levels of PKCθ protein, with PRKCQ-targeting shRNA viral supernatant, did not inhibit their growth in 3-D cultures (Additional file 1: Figure S2).

Consistent with the phenotypes observed in the in vitro cultures, PRKCQ/PKCθ downregulation impaired growth of triple-negative breast primary tumor xenografts (Fig. 5e). Downregulation of PKCθ in MDA-231-Luc-D3H2LN cells using two independent shRNA vectors (90 and 54) inhibited growth of primary tumor xenografts following subcutaneous injection into the flanks of nude mice. These data show for the first time that endogenous PRKCQ is necessary for the in vivo growth of human TNBC, and that PRKCQ plays a unique role that is not redundant with those of other PKC isoforms.

**Fig. 6:**
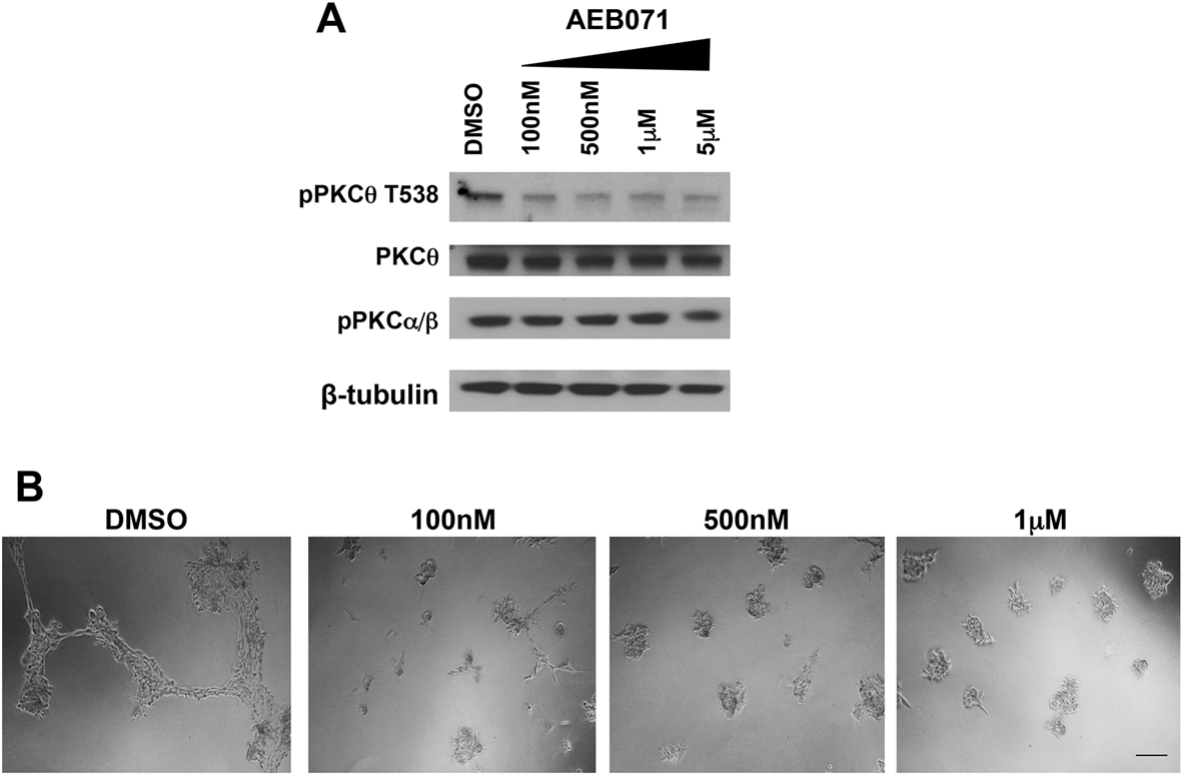
Treatment with AEB071 inhibits protein kinase C (*PKC*)θ activity and impairs growth of triple-negative breast cancer cells. **a** MDA-231-Luc-D3H2LN cells were treated with AEB071 at the indicated concentrations for 24 hours. Cells were lysed and probed with antibodies that recognize autophosphorylated PKC isoforms. **b** MDA-231-Luc-D3H2LN cells were treated with the indicated concentrations of AEB071 in 3-D Matrigel™ cultures for 7 days with refeeding every 2–3 days. *Scale bar* indicates 200 μM. *DMSO* dimethyl sulfoxide

Several PKC inhibitors have been developed. Although none are currently completely specific for PRKCQ/PKCθ, AEB071 has demonstrated selective activity against novel and classic PKC family members [29]. We confirmed that treatment of MDA-231-Luc-D3H2LN cells with AEB071 inhibited PKCθ autophosphorylation at Threonine 538, even at doses as low as 100 nM (Fig. 6a). In contrast, AEB071 treatment of TNBC cells at the doses evaluated did not affect the activity of PKCα/β2, both classic PKC’s, as assessed by autophosphorylation at Threonine 638/641 (Fig. 6a). Treatment of MDA-231-Luc-D3H2LN cells with AEB071 impaired growth in 3-D Matrigel^TM^ cultures and prevented invasive branching at doses that inhibit PKCθ autophosphorylation, thus phenocopying the effects of PRKCQ shRNA expression (Fig. 6b). These data further support the importance of PKCθ kinase activity in the oncogenic activity of TNBC cells, and the potential for clinical translation of PKCθ kinase inhibitors as a therapeutic option for patients with TNBC.

## Discussion

The functions of PKC family members are varied and diverse. PKC expression or activity is detected in cancer cells and for this reason, inhibitors of the PKC family have been developed and evaluated as potential cancer therapeutic agents. However, their use in cancer therapy has been limited. One potential reason may be the lack of biomarkers and the potential isoform-specific functions of PKC enzymes. While many of the PKC enzymes are associated with pro-tumorigenic activities, there is also evidence that supports possible tumor-suppressive roles for some PKC family members. For example, mutations detected in PKC enzymes in patient tumors have been reported to have growth-inhibitory properties in vitro [30]. Therefore, the precise functions of specific PKC isoforms in human cancer need to be clearly elucidated for optimal development of PKC isoform-targeted therapeutic agents.

PRKCQ/PKCθ has been extensively studied in the context of hematopoiesis and immunity, and is required for the development and maturation of specific T cell subsets. PRKCQ also plays a role in Notch-driven T cell leukemia [31]. However, its function in epithelial cancer has been relatively underexplored. PRKCQ is expressed or activated in a subset of GIST, uveal melanoma, and breast cancers [17, 19, 20, 32]. Here, we show that PRKCQ is sufficient to promote oncogenic activities such as anoikis resistance and growth-factor-independent migration and proliferation. Our studies are also the first to demonstrate the isoform-specific requirement for endogenous PRKCQ in the in vitro and in vivo growth of a subset of human TNBC cell lines. PRKCQ/PKCθ expression is relatively greater in human triple-negative tumors compared to other subtypes, further supporting PRKCQ/PKCθ as a candidate therapeutic target for this subtype.

Our studies highlight the critical role of PKCθ kinase activity in tumorigenic phenotypes; kinase activity is required to enhance growth-factor-independent cell-cycle progression of MCF-10A cells via Erk/MAPK activity and Rb phosphorylation, and inhibition of PKCθ kinase activity impairs the growth and invasiveness of TNBC cells. These results support the possibility of targeting PRKCQ kinase activity as a therapeutic approach for a subset of TNBC.

Although our studies are the first to show a critical role for endogenous PRKCQ in the growth and survival of a subset of human TNBC cells, our data complement previous work that suggested a role for PRKCQ in ER-negative breast cancers. Belguise et al. report that PRKCQ suppresses ER transcription via Akt activation and Forkhead inhibition, and PRKCQ is required for c-Rel-driven, ER-negative development of a mouse mammary tumor [20]. PRKCQ also promotes the stabilization of Fra-1, a member of the Fos transcription factor family in ER-negative breast cancer [21].

Interestingly, in addition to its role in regulating signaling in ER-negative breast cancer, PRKCQ has been shown to localize to the nucleus where it directly interacts with chromatin complexes to induce expression of genes associated with an epithelial-to-mesenchymal transition, and markers associated with a cancer stem cell phenotype [23]. Kinase activity of PKCθ was required for this induction of mesenchymal gene expression. These data and our own results showing the kinase-activity-dependent activation of Erk/MAPK signaling by PRKCQ highlight the potentially multiple ways by which PRKCQ kinase activity may promote oncogenic activity in TNBC cells via signaling and transcriptional mechanisms in specific subcellular compartments.

The recent synthesis of PRKCQ/PKCθ small molecule kinase inhibitors raises the attractive possibility of using these to therapeutically target PRKCQ in TNBC. These inhibitors are highly specific for PRKCQ, relative to other PKC isoforms, and have been developed for the treatment of autoimmune or inflammatory disease [33]. The kinase dependency of PRKCQ oncogenic activity supports the evaluation of these inhibitors in the context of breast cancer models. Given the expression of PRKCQ in the cells of the immune system, the effect of PRKCQ inhibition on host immunity must be considered. However, as PRKCQ-/- mice appear to maintain normal immune responses to most bacterial and viral pathogens, it is unlikely that PRKCQ inhibition will significantly compromise the host defense mechanisms reviewed in [2]. Interestingly, PRKCQ is specifically required for the maturation and development of Th17 cells, which have been implicated in autoimmune diseases, such as multiple sclerosis and arthritis [14, 15]. Although the role of Th17 cells in cancer development or progression is controversial, IL-17 and other cytokines secreted by Th17 T cells have been shown to promote tumorigenesis [34]. The use of these isoform-specific PRKCQ inhibitors could provide unique insights into the interaction between triple-negative tumor cells, their immune microenvironment and systemic immune responses.

## Conclusions

Our studies support PRKCQ/PKCθ as a therapeutic target for TNBC. Enhanced PRKCQ expression is sufficient to promote anoikis resistance, migration, and EGF-independent growth via kinase-activity-dependent stimulation of Erk/MAPK. Inhibition of PRKCQ, either by downregulation of expression or inhibition of kinase activity, suppresses TNBC growth, highlighting the potential of PRKCQ small molecule inhibitor treatment as a therapeutic strategy.

## Additional file

Additional file 1: Figure S1. PRKCQ downregulation in MDA-231-LucD3H2LN cells results in G2-M arrest. Triple-negative MDA-231-LucD3H2LN cells were infected with PRKCQ shRNA lentiviral supernatant for 48 hours. Cells were fixed and stained with PI, and cell-cycle profile analysis was performed using flow cytometry. Results from four independent experiments were averaged. Figure S2 PRKCQ shRNA has no effect on growth of MDA231 cells that do not express detectable PKCθ protein. MDA231 cells expressing empty vector (EV) or PRKCQ shRNA (54) were grown in 3-D cultures for 7 days. (PDF 573 kb)

## Abbreviations

3D: Three-dimensional
Bcl2: B cell lymphoma 2
Ca+: Calcium
DMEM: Dulbecco’s modified Eagle’s medium
DMSO: Dimethyl sulfoxide
EGF: Epidermal growth factor
ELISA: Enzyme-linked immunosorbent assay
ER: Estrogen receptor
Erk: Extracellular signal-related kinase
FBS: Fetal bovine serum
GAPDH: Glyceraldehyde-3-phosphate dehydrogenase
GIST: Gastrointestinal stromal tumors
Her2: Human epidermal growth factor receptor 2
IL: Interleukin
MAPK: Mitogen-activated protein kinase
MEK: Mitogen-activated protein kinase kinase
MEM: Modified Eagle’s medium
NFAT: nuclear factor of activated T-cells
PI: Propidium iodide
PR: Progesterone receptor
PRKCQ: Protein tyrosine kinase C theta isoform
Rb: Retinoblastoma
RNA: ribonucleic acid
RPMI: Roswell Park Memorial Institute
shRNA: Short hairpin RNA
TCGA: The Cancer Genome Atlas
Th: T helper
TNBC: Triple-negative breast cancer
