# Supplementary figures and images for: PRKCQ promotes oncogenic growth and anoikis resistance of a subset of triple-negative breast cancer cells
Source: Breast Cancer Res. 2016 Sep 23;18:95. doi: 10.1186/s13058-016-0749-6 (PMC5034539; doi:10.1186/s13058-016-0749-6)

Supplemental Fig 1

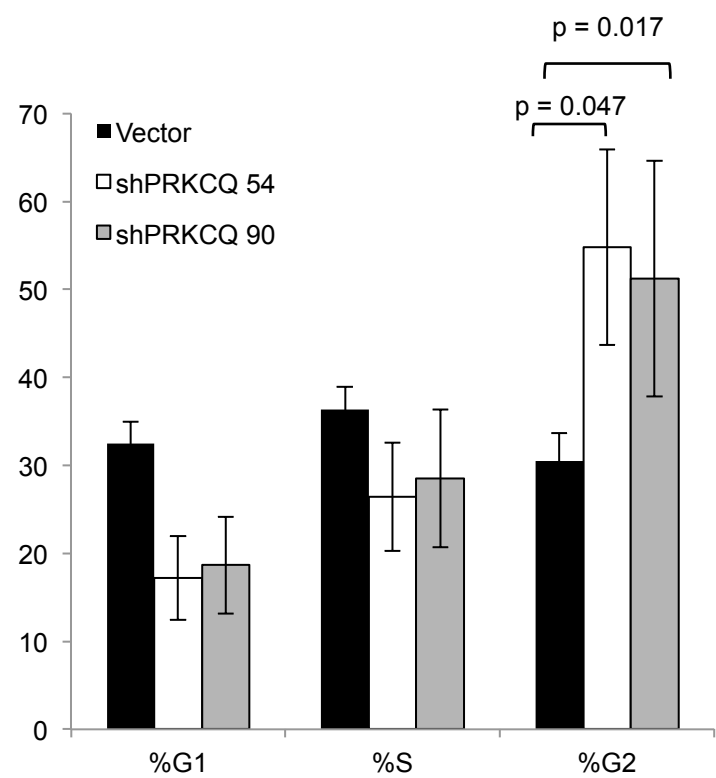

## Supplemental Fig 2

### MDA-231

**EV**

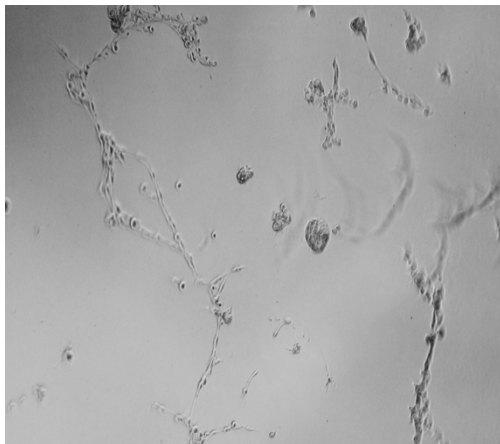

**shPRKCQ54**

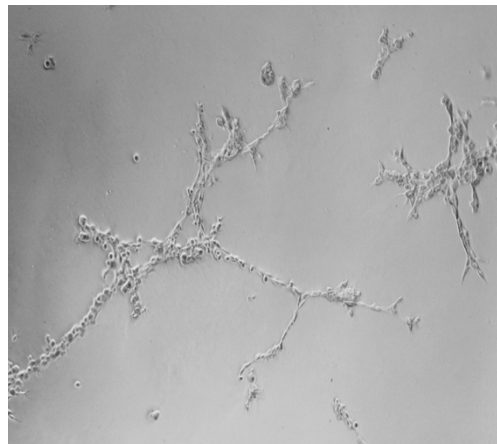

Supplement: Additional file 1: Figure S1. — PRKCQ downregulation in MDA-231-LucD3H2LN cells results in G2-M arrest. Triple-negative MDA-231-LucD3H2LN cells were infected with PRKCQ shRNA lentiviral supernatant for 48 hours. Cells were fixed and stained with PI, and cell-cycle profile analysis was performed using flow cytometry. Results from four independent experiments were averaged. Figure S2 PRKCQ shRNA has no effect on growth of MDA231 cells that do not express detectable PKCθ protein. MDA231 cells expressing empty vector (EV) or PRKCQ shRNA (54) were grown in 3-D cultures for 7 days. (PDF 573 kb) [file 13058_2016_749_MOESM1_ESM.pdf]
